# Proving of Amm. Carb.

**Published:** 1881-07

**Authors:** Leila A. Rendell

**Affiliations:** New Haven, Conn.


### PROVING OF AMMONIUM CARBONICUM.

LEILA A. RENDELL, M. D., NEW HAVEN, CONN.

I TOOK one dose of *Amm. Carb.* 1 M. (Fincke), and on the 27th day took a dose of 10 M. (Fincke). On the 72d day, being completely exhausted by the severity of the symptoms, I having become very thin and weak, I took a dose of *Lachesis* C.M. (Fincke). All the symptoms were greatly ameliorated by next day, and most of them soon ceased.

The proving is arranged, for convenience of reference, in Hahnemannic order. A few symptoms, which the proving removed in me, are marked °. The numbers in parenthesis, following the symptoms, signify the days on which they occurred, reckoning from the *first* dose.

### MIND AND DISPOSITION.

Think of everything which others have done to displease me; lie awake all night to lay plans to talk to them about it, but forget it in the morning (4 to 9).

Diminished thinking power (15 to 27).

Great forgetfulness and inability to collect my senses (15 to 27).

Use wrong words in writing or talking (15 to 27).

Very forgetful; make mistakes in writing or speaking (55 to 62).

Aversion to work, not disposed to do anything (61 to 65).

Disposition to weep in evening (61 to 64).

Full of care and gloomy anxiety; thoughts about unpleasant occurrences of former times crowd upon the mind and torment me (15 to 28).

Sad and desponding as if some accident were impending (15 to 27).

10. Loathing of life; everything looks so dark, cannot think of anything worth living for (15 to 27).

Great dislike to go out of doors; neglect important things on that account (15 to 27).

Gloomy, depressed, with feeling of impending danger and trouble, with sensation of coldness when thinking of it, and the lips look blue (28 to 30).

Utter dejection of mind, listlessness and lethargy (28 to 30).

Anxiety, with inclination to weep (28 to 30).

Hearing others talk, and talking myself, makes me very weary and nervous; cannot bear it; must get away from it and people (28 to 30).

Anxious concern about my sickness; when my friends try to cheer me up, I say to them: "I know I will never amount to anything, for I can never use my brain again, and will never advance another step in my profession;" the tears will come, I cannot keep them back (28 to 30).

Sad, despondent, homesick; tears run down my cheeks in evening and at night; have lost all self-respect, for I cannot do anything right; feel I ought not to prescribe for a patient, am sure I do not know enough, despise myself because I do not know more; brain feels so thick, cannot get a thought through it, it will not act; when I see a patient come in the office I feel so badly because I know I cannot do well for them, so that I am all in a tremble and can hardly speak, the perspiration runs down my back; I tremble so, I lose the powder out of the paper when trying to do it up; I try to conceal these symptoms, I am so ashamed of them; cannot see anything good in myself, feel so out with myself, with utter contempt of self, but see good in every one else, even my worst enemy; will stand up for them, and point out some good in them, and make excuses for them; constantly admiring the good qualities I see in others, but think I am all bad, and that others think so: these symptoms lasted till end of proving (53).

### HEAD.

Vertigo, with pressure in centre of brain; was obliged to take hold of something to keep from falling (14).

Head feels dull, muddled and stupid (15 to 27).

Confusion of head (28 to 30).

20. Frequent giddiness, as if the surroundings were turning with me in a circle; worse in morning, but lasting the whole day; worse at night on moving the head (32 to 40).

The same; worse in morning after rising, and at night when moving head (48 to 52).

Sensation of looseness of the brain, as if it fell to the side toward which I leaned (32 and 63).

Congestive headache (34 to 39).

When stooping, sensation as if the blood accumulated in the brain (34 to 40).

Bursting headache when moving about (66).

Intense heat in head and face (47).

Pain in top of head, extending down to both ears (49).

Heaviness and beating in forehead after dinner (50 to 54).

Sense of oppressive fullness, pulsating as if forehead would burst (55 to 59).

Sensation as if the right temple were drawn so tight that it would snap; very painful (50).

30. Pimples on forehead, tip of nose, chin and temples (15 to 27).

Severe itching on scalp, worst on occiput (28 to 30).

Scalp and hair painful to touch (29 to 30).

Drawing pains in periosteum of forehead, in morning (30).

Pimples on scalp, burning, stinging, smarting and itching (35 to 59).

Severe itching on scalp, especially on occiput; eruption resembles that produced by using croton oil; very tender (38 to 50).

Pustular eruption on forehead, cheeks and chin (50 to 55).

Pimples on scalp, commencing on crown, and extending all over scalp and down back of neck and upper parts of shoulders; these somewhat resemble small-pox, having the shot-like feeling under the skin, the lymph-indentation and the maturating; the itching was intense, and the hair fell out very badly; my head and neck became one mass of oozing matter, having a very unpleasant odor, something like india-rubber, and sweetish, and very sticky. On 64th day I felt a crawling as if there were lice on it, but thought it only a sensation; in the afternoon it was so troublesome and real that I used a fine comb, and discovered over 50 very small lice; the smell and the idea of lice made me gag and vomit; I washed my head twice a day, and had been doing so during the eruption, to subdue the heat and itching, so that it did not arise from want of cleanliness. I continued the washing and combing twice a day, but it was impossible to get rid of them as long as there were any new pimples breaking out; the last were on my neck and shoulders, and the lice seemed to crawl from them the same as from my head, I could pick them off (57 to end of proving).

### EYES.

Dimness of sight; and when making an effort to see, objects seem double (15 to 27).

Short sight (15 to 27).

40. Aversion to light (28 to 30).

Agglutination of lids in morning; copious lachrynation during day (10).

Pressure about lids on waking up; pressure in eyes, and intolerance of light on waking, and in evening when lying down (15 to 27).

Smarting in eyeballs, and itching of margins of lids; worse at night and very early in morning (32 to 40).

Eyelids adhere in morning, have to pick them open; when open, sensation of a deep, perpendicular gash in outer side of left eye, entering eye at junction of the cornea and sclerotic, with smarting and burning (41 to 44).

Feel as if the upper lids were too short transversely, can only open them half-way; heat and dull sore aching pain in upper back part of left eyeball; they feel very tired because I have to make such an effort to keep them open; my eyes are only half open all the time, and look and feel as if they had been strained, worse from movement (41 to end of proving); smarting of eyes, and stinging in margins of lids (55 to 57); smarting in eyes (57 to 58).

Have to sit down after dinner and sleep a few moments, otherwise eyes very painful and sore during remainder of day (15 to 27).

### EARS.

Indistinct hearing (50 to 53).

50. Intolerance of noise, particularly hearing people talk (15 to 27).

Right ear, which was deaf seven years ago, is painfully sensitive to noise; a strong sound was so painful to it, it made me tremble all over (15 to 27).

Sensation as if a bird were fluttering its wings close to left ear; later in the day, felt as if there were a large insect in the ear fluttering its wings (50).

Drawing feeling through the whole of both internal ears (50).

### NOSE.

Sneezing (28 30).

Frequent sneezing early in morning (11 to 15).

Half an hour after taking the second dose, profuse watery discharge from nose, and at noon, each day afterward, for about an hour (27 to 41).

Burning water runs out of nose, worse when stooping (37 to 41).

Stoppage at nose at night (37 to 41).

Scabs in nose in morning (37 to 41).

60. Pustules on nose (46 to 47).

### FACE.

Contraction of skin of face and forehead; sense of stretching in face, causing me constantly to rub eyes and face (15 to 27).

Tension of face, nose and lips, which are swollen early in morning (15 to 27).

Red cheeks; head, face and eyes feel so hot that it starts the tears (54).

Face very much swollen in morning; looks very pale; eyes dull, with dark circles round them; also little bags under eyes (65 to 71).

During day looked pale, but towards evening my face was very much flushed, with fever, lips blue, eyes dull and aching; aching of the whole body more intense than ever; my friends say I am going to have a fever (28 to 30).

Heat in face with red cheeks (34 to 40).

At 3 P. M., a tired feeling in face, particularly round eyes; "crows' feet" around eyes; loss of control of facial muscles; they feel weak and contracted, as if they could not act; face lacks expression; deep lines in face: these symptoms last all the evening (35 to 40).

Sore on left side of lower lip, itching, stinging and burning, very painful; not a fever-sore, but is convex, hard and looks bluish (34 to 40).

Lips very much swollen, smart and sting, feel as if they would crack open when moved; upper lips so swollen that it is rolled up and nearly touches the nose (37 to 41).

70. Upper lip full of fine cracks, with deep cracks in corners of mouth (54 to 56).

Pain and swelling of sub-maxillary glands; they feel tense upon the mouth being opened or moved (15 to 27).

### TEETH.

The lower front teeth feel too long, and as if they were not my own, but of china; constant uneasy feeling in the teeth; am all the time inserting toothpicks between them, and pressing them apart; they feel lame and sore (13 to 19).

A sac, the size of a pea, formed above left upper first bicuspid, hung down on a peduncle, looking like flesh; on opening it, it bled freely and relieved toothache, which had been very bad all day (35).

Teeth feel too long and dull (37 to 40, and 55 to 60).

Stitching pain in molars when biting, must use incisors only (38 to 39).

When masticating, decayed second left lower bicuspid pains more (38, and 59 to 60).

Severe toothache (66).

Pinching pains, especially in molars, worse when masticating or touching the decayed teeth with the tongue (55 to 60).

Gums feel sore and tender, easily bleed (54 to 63).

Gums feel swollen, easily bleed (56 to 64).

Throbbing pain in gums and roof of mouth, worse from stooping (59).

Pressing the teeth together sends a shock through head, ears and nose (45 to 69).

### MOUTH.

Bad smell from mouth perceived by myself (15 to 27).

Acid taste in mouth, worse from drinking milk; milk becomes so offensive, I do not even want to see it (15 to 27).

Great dryness of mouth and throat, but no thirst (28 to 29).

Redness and inflammation of inner mouth and gullet; pain as if raw (33 to 35).

Sensation as if mouth were swollen, with great dryness and thirst (54).

Fever blisters on front part of roof of mouth; after breaking, leave a purple scar (55 to 60).

Evening and night, profuse saliva troubles me very much; when talking, it runs out of corners of mouth (54 to 56).

90. Have to spit often (52 to 59).

Vesicles on tongue at tip and edges, hindering eating and talking (38 to 46.)

THROAT.

Great dryness of throat (37 to 41).

Sensation as if there were feathers in throat; lasting to end of proving (42).

Dryness and burning of throat, with strings of phlegm, which cause constant swallowing, but cannot break them up; they string down so long without breaking that they choke me; a very unpleasant taste to it (47 to 51).

Pain in throat as if right tonsil were swollen, painful to swallow (55 to 60).

Great dryness of throat and hoarseness; catarrh; dry cough, worse at night; sensation of a feather down in throat (59 to 61).

STOMACH.

Unconquerable appetite for sugar (31 to 34, 36 to 40, 54 to 56).

Frequent sour eructations (15 to 27).

Nausea, vomiting a cup of cocoa I had forced down (28 to 30).

100. Complete loss of appetite, cannot get the food down; feel very weak, cannot sit up, obliged to leave the table and lie down (28 to 30).

No appetite, except for cold food (42 to 46).

°Before taking *amm. carb.*, I would not eat white bread, or if I did, I would not digest it, but would vomit it; during the proving I could eat it, and can do so now, after the proving, without any bad effect.

Stomach feels full and trembling (15 to 27).

Uneasiness in stomach, with pressure in stomach and forehead, worse after eating (15 to 27).

Painfulness of stomach when pressed upon; the clothes press upon it (15 to 27).

Oppressive weight at stomach-pit (15 to 27).

Pinching and rumbling in stomach-pit (15 to 27).

Heat of stomach which extends to bowels, as from drinking strong wine (15 to 27).

### Abdomen.

Weight in abdomen (15 to 27).

110. Weight of abdomen; distension of abdomen; rumbling and shifting of flatulence in abdomen; griping with a great deal of flatulence in abdomen; emission of a great deal of flatulence in afternoon, evening and night, causing a tender pain (9 to 16).

Painful concussion in lower part of abdomen when stooping; felt as if all the internal organs were loose and very sore (11 to 15).

Pressure upon a spot as large as a quarter dollar, an inch above and to left of umbilicus, as if there were a hard substance there, and as if the tissues around it were bound down by it; every movement causes a pulling sensation from that spot; very tender pain (14 to 22).

Sudden painful contraction of bowels as far as region of stomach (15 to 27).

Contracting, pinching pain in abdomen, very severe; first the upper then the lower part (15 to 27).

Violent pinching, contraction and rumbling in abdomen (15 to 27).

Crampy feeling and sense of obstruction in abdomen (15 to 27).

Tender smarting pain an inch above and to left of navel, worse from pressure and movement (39 to 50).

Drawing together in left lower bowels (45 to 53).

Stitches in right hypochondrium (45 to 47): ditto, worse from movement (14 to 16).

120. Eruption on abdomen, very thick and red (31 to 52).

### Stool and Rectum.

Constipation; hard balls, hard to press out (6 to 9; 36 to 40).

Four loose passages every day (41 to 45).

Diarrhœa, with colic low down in abdomen, early in morning and tenderness during the day, diarrhœa with cutting in abdomen; evacuations with constant tenesmus during stool, and remaining half an hour after stool; the tenesmus is felt only in upper back part of rectum (14 to 20).

Deep cracks in perineum, very painful when urinating, defecating, or when wet (55 to 60).

Itching of anus (14 to 18)

Tenderness of upper back part of rectum (41 to 60).

### URINE.

Violent pressure of urine upon bladder, with cutting and constant urging even at night, with diminished emission of urine, accompanied with burning (15 to 27).

Obliged to rise four times every night to urinate; frequent micturition during day, light colored and profuse, foams very much, the foam remaining on it when the vessel is emptied; on testing, find it loaded with albumen; some days a whitish sediment in urine; pressure on bladder with cutting pains (43 to end of proving).

Roused from sleep with urgent desire to urinate (15 to 27).

130. Urine very light yellow, frequent, increased in quantity, leaving a pinkish sediment, which adheres to the vessel (15 to 27).

Urine very scanty and dark, causing burning when passing it (31 to 34).

Urine scanty, thick and dark (62 to 64).

Urine dark, thick, scanty and frequent (4 to 7).

### SEXUAL ORGANS.

° Swelling and pain in breasts before menses, were removed by the proving.

° Used to suffer very much during menses; this has ceased since the proving, but I have congestive headache, instead.

Congestive and menstrual headache, with pinching pain in centre of abdomen (55).

Quick darting and cutting pain in uterus, from below upwards, lasting all day, worse from movement (33).

Severe cutting pain in left ovary, darting down to groins (47).

Aching tensive pain in left ovary, extending to left groin and left thigh; worse from movement (66, 67).

### CHEST.

140. Sound of cracking in chest (48 to 53).

Contraction in middle of chest, either when taking a long breath or from natural breathing; the place aches when pressing upon it, as after a blow (15 to 27).

Tickling in throat and bronchial tubes, as if filled with dust, worse from taking a long breath (37 to 41).

Stitches in right side of chest, when stooping, walking or rising in bed (48 to 52).

On taking a long breath, the whole chest and lungs feel weak and bruised, very painful; makes me give a dry, hacking cough, which causes a pain through to back between shoulders, and a smarting, raw sensation in bronchial tubes; lungs feel so weak all the time, it tires me to talk; get out of breath when talking, breathing short all the time (65 to 70).

Talking difficult at times, like as from weakness of the parts (48 to 52).

Shortness of breath and palpitation from the slightest movement; even rousing up in bed produces it (28).

Difficult breathing, causing short, dry cough (30 to 36).

Cough, short, asthmatic, from irritation in larynx, with painful sensation of spasmodic contraction of chest (51, 52).

Cough, with asthma; evening in bed (59).

150. Two or three little coughs early in morning, and coughed up a large piece of firm, yellow phlegm, tasting sweet, and remaining in form after spitting it out (35 to 65).

At 11·30 A. M., when out walking, felt as if blood passed into heart but did not pass out; the heart felt so full as if it would burst; severe tensive pain in heart, felt as if the muscles of heart tried to act, but were so rigid that they could not; very numb all over; lips looked blue, with white margins for about an inch around them; face pale; eyes looked dull; head feels anæmic; in about fifteen minutes a severe pain in back of throat; inclination to swallow, but when trying, the muscles seem rigid and refuse to act; the pain gradually extends down the œsophagus to stomach; breathing all the time very short and difficult; all the muscles of respiration seem rigid, causing a tensive pain when using them; the pain extends to stomach and liver, and after about ten minutes, subsides in throat and stomach, but not in heart, which grew worse. I could not walk or stand, I staggered so; anæmia in head worse; numb all over and cold; looked and felt very ill; in about half an hour the pains extended from liver to between scapulæ, making breathing even more difficult; from scapulæ to teeth, all aching at the same time; then passed to right ear. Desire to take a long breath, but cannot; panting breathing; cannot lie down. These pains lasted till 2.30 P. M., then became less, and I commenced yawning and stretching; look pale, and face and eyes have not the slightest expression; eyelids heavy and droop; feel weak and lifeless; yawning, stretching, numbness and drawing in muscles continue till late in evening, when the heart commences to contract better; there is a sensation as if blood were swashing in brain in top part of head; during these symptoms, I look and feel very sick. Had these symptoms every day till end of proving, growing more severe (40).

Palpitation of heart (66).

A severe, throbbing, cutting pain in apex of heart (40).

Palpitation of heart, very violent (60 to 65).

Awake in night with a dull, heavy pain in heart, passing from apex to base; must sit up; feel as if heart had stopped beating (61 to 64).

Small pimple on sternum, which, when touched, feels as if there were a splinter in it (37 to 40).

### BACK.

Pains in small of back, increased by motion and walking; also, when stopping; sensation as if the muscles were not strong enough to support the body, which constantly threatens to fall forward (15 to 27).

Bruised feeling in small of back (15 to 27).

Drawing pain in small of back, extending into legs and loins, when at rest (15 to 27).

160. Gnawing pain in small of back (15 to 27; 62 to 72).

Violent pain in small of back, with great coldness (37).

Aching pain in kidneys; makes me feel weak and sick all over; eyes look dull, sight is indistinct, eyeballs smart and itch (64 to 66).

Pain between scapulæ (38 to 46).

Pain in back and loins, worse from rest, relieved by lying on abdomen (30 to 49).

Pressure in back (15 to 27).

Drawing all along back, beginning at nape, causing stiffness when turning head and severe drawing in nape (15 to 27).

Enlarged gland back of neck, just at edge of hair, very painful indeed (64 to 72).

### UPPER EXTREMITIES.

Burning, stinging and redness of a wart on back of left hand near wrist; constantly picking at it until I picked it off; it did not bleed and has not returned (2 to 5).

Hands look blue, and veins distended after washing in cold water (50 to 56).

170. Fingers go to sleep (57 to 60).

Drawing pain and cramp in right upper arm and shoulder, drawing arms backward, cannot bring it forward; numbness extends down arm to hand; lose all power in it; cannot hold anything, drop everything I may have in my hand; restless, want to move arm constantly; worse from hanging arms down; better from holding arms up, with elbows bent; usually comes on from noon to 3 P. M.; always while walking (3 to 21; also, much more severely 34 to 40).

Tearing in shoulders (15 to 27).

Tearing in left shoulder, extending to chest, very marked (15 to 27).

Pain, as if bruised, in left shoulder, when at rest (15 to 27).

Lameness and drawing in left arm from axilla to wrist (15 to 27).

Right arm feels as if it weighed a hundred-weight, and is without strength (15 to 27).

Weight and lameness in right arm; have no power in it; must let it hang (37).

LOWER EXTREMITIES.

Ball of left great toe feels hot and bruised; worse in morning (32 to 40).

Sensation as if left great toe and two adjoining toes unjointed when rising from a chair and trying to walk; sometimes so painful as to start the tears; sometimes lasting about an hour, at others only a few minutes (18 to 20).

180. Ball of left great toe painful and hot, felt when walking on it as if I were stepping on a sharp knife (19, 20).

Tearing in ankles and bones of feet, ceasing when warm in bed (37 to 59).

Pain in outer side of left lower leg, restless, but worse from movement; feel as if the muscular part had grown together, without any movement of the fibres on each other; when trying to move leg, can feel the pulling on periosteum as if it would be torn from the bone; if I continue to try to move it, the muscles will not yield, and there is a burning, smarting, gnawing pain in periosteum (9 to 14).

Severe cutting drawing pain in inner side of right knee, in periosteum; makes me cry out; cannot move it; lasting about 20 minutes (11).

Boring and drawing pain in knees (46 to 51).

Great lassitude of thighs and legs (15 to 27).

Sense of scraping upon bones of thighs and legs at intervals (15 to 27).

Sudden and great weakness in lower limbs (15 to 27).

### GENERALITIES.

Constant stretching of arms, legs and feet (15 to 27).

All my limbs pain me at night, with gnawing pain in small of back; have to turn myself slowly in bed, because motion gives me pain (15 to 27).

190. Cracking in joints when walking (15 to 27).

Hands and feet go to sleep when sitting (15 to 27).

Great exhaustion and aching soreness over whole body (28).

Heaviness of all internal organs (29, 30).

Feel as if I were going to have a severe illness; cannot sit up, and look very ill (29, 30).

Feel as if I were rigid all over; a set feeling in eyes; heavy, dull ache in head; seem as if I would lose myself in a stupor; stop talking and sit with closed hands in a tight grip; do not answer patients; with a great effort, got up and walked across the room; felt somewhat relieved, but the feeling remained all the afternoon (54).

Ebullition of blood at night—seems as if the heart and veins would burst (55 to 60).

Very sensitive to cold air out of doors (62 to 66).

° Since an illness six years ago, the muscles and tissues have seemed to be so bound together, not moving freely on each other as they used to; could not lie on back, there seemed to be such a tightness of the flesh from chest to abdomen; can now lie on back with ease, and the muscles of body seem to act freely and naturally; can walk with much greater ease since taking the *Amm. Carb.*

### SLEEP.

Light sleep at night; every little noise wakes me (15 to 27).

200. Could not sleep at all during night nor next day (28 to 30).

Restless, unrefreshing sleep, tossing about (39 to 53).

Dreams vivid; dream of having nose-bleed (37 to 54; 57 to 59).

Dreams vivid, lewd and full of danger (41).

### FEVER.

High fever, great internal heat; feel as if I were burning and drying up internally, in body, legs and arms (28 to 31).

Continuous night-sweat (68).

### SKIN.

In afternoon, body bright-red like scarlatina; the redness remained for nine days (29).

Violent itching; after scratching, burning blisters appear (52 to 58).

Thick dead cuticle peels off from back of ears, groin and between toes (52 to 62).

Itching and stinging of skin keeps me awake (64 to 72).

Much general itching of whole body during entire proving.

